# Coexistence of Growth Hormone Deficiency and Pituitary Microadenoma in a Child with Unique Mosaic Turner Syndrome: A Case Report and Literature Review

**DOI:** 10.3390/diagnostics10100783

**Published:** 2020-10-04

**Authors:** Eu Gene Park, Eun-Jung Kim, Eun-Jee Kim, Hyun-Young Kim, Sun-Hee Kim, Aram Yang

**Affiliations:** 1 Department of Pediatrics, Incheon St. Mary’s Hospital, College of Medicine, The Catholic University of Korea, 56, Dongsu-ro, Bupyeong-gu, Incheon 21431, Korea; eugene.park@catholic.ac.kr; 2 Samsung Medical Center, Department of Laboratory Medicine and Genetics, Sungkyunkwan University School of Medicine, 81 Irwon-ro, Gangnam-gu, Seoul 06351, Korea; ej1219.kim@samsung.com (E.-J.K.); eunjee.kim@samsung.com (E.-J.K.); hyuny.kim@samsung.com (H.-Y.K.); sunnyhk.kim@samsung.com (S.-H.K.); 3 Department of Pediatrics, Kangbuk Samsung Hospital, Sungkyunkwan University School of Medicine, 29 Saemunan-ro, Jongno-gu, Seoul 03181, Korea

**Keywords:** Turner syndrome, mosaicism, ring chromosomes, growth hormone deficiency, pituitary microadenoma

## Abstract

Turner syndrome (TS) is a genetic disorder with phenotypic heterogeneity caused by the monosomy or structural abnormalities of the X chromosome, and it has a prevalence of about 1/2500 females live birth. The variable clinical features of TS include short stature, gonadal failure, and skeletal dysplasia. The association with growth hormone (GH) deficiency or other hypopituitarism in TS is extremely rare, with only a few case reports published in the literature. Here, we report the first case of a patient with mosaic TS with complete GH deficiency and pituitary microadenoma, and we include the literature review. During the work-up of the patient for severe short stature, three GH provocation tests revealed peak GH levels of less than 5 ng/mL, which was compatible with complete GH deficiency. Sella magnetic resonance imaging showed an 8 mm non-enhancing pituitary adenoma with mild superior displacement of the optic chiasm. Karyotyping revealed the presence of ring chromosome X and monosomy X (46,X,r(X)/45,X/46,X,psu dic r(X;X)), which indicated a mosaic TS. It is important to consider not only chromosome analyses in females with short stature, but also the possibility of the coexistence of complete GH deficiency accompanying pituitary lesions in TS. In conclusion, the present study reports the first case of GH deficiency and pituitary adenoma in a patient with rare mosaic TS, which extends the genotype–phenotype spectrum for TS.

## 1. Introduction

Turner syndrome (TS) is a genetic disorder occurring in females caused by the partial or complete absence of one of the X chromosomes. The condition affects approximately 1 in every 2500 females and requires a chromosomal analysis for definite diagnosis [1]. Short stature and hypergonadotropic hypogonadism are the principal features of TS [2,3]. Patients with TS are also susceptible to numerous other medical conditions, such as endocrine and metabolic disorders, autoimmune disease, and cardiovascular disease [4]. Multiple karyotypes including 45,X haploinsufficiency, 45,X with mosaicism, or X chromosome anomalies are associated with variable presentations along the TS phenotype spectrum; individuals with 45,X monosomy typically have the most severe phenotype [5].

Mosaic TS are subcategorized according to whether the second cell line contains a whole or part of a sex chromosome. In a study by Jacobs et al. [6], 16% of the 84 cases with TS had a standard karyotype of 45, X and a second cell line containing a ring chromosome X. The phenotypic variability of these mosaics is largely dependent on the size of the ring and the presence of a functioning *XIST*.

Patients with TS tend to have short stature and high body mass indices [7], but most often do not have growth hormone (GH) deficiency [4]. Females with TS make GH naturally in the pituitary gland, but their bodies do not use it appropriately. GH provocation tests are generally not indicated in TS unless the growth velocity is extremely low for the age and sex. Thus, the concurrent occurrence of GH deficiency and TS is a very rare condition. Moreover, the association of TS with hypopituitarism is also an uncommon finding [8].

To the best of our knowledge, there have been no previous reports of concomitant GH deficiency and structural pituitary abnormalities in TS. Here, we report the first case of the coexistence of GH deficiency and pituitary microadenoma in a TS patient.

## 2. Case Presentation

A female aged 13 years and 3 months visited the pediatric endocrinology clinic due to short stature. She was born at term via vaginal delivery weighing in at 2.5 kg and had no history of perinatal problems. The patient was the second child of non-consanguineous, healthy parents. Her medical history was unremarkable and did not include any head trauma, seizure, or infection of the central nervous system. No specific family history was found. The paternal and maternal heights were 169 and 163 cm, respectively, and the midparental height of 159.5 cm was within the normal range.

Ethics Statement: This study was approved by the Institutional Review Board of the Kangbuk Samsung Hospital and conducted according to the Declaration of Helsinki ethical principles (IRB 2019-11-051-001). Parental informed consent was obtained in accordance with institutional review board standards.

The patient was 133 cm (−3.4 standard deviation scores (SDS); 50th percentile in growth curves for TS (Figure S1)) in height with a growth velocity of less than 4 cm/year. She was 38.1 kg (10th percentile) in weight, and 21.6 kg/m^2^ (79th percentile) in body mass index. The physical examination was unremarkable. The sexual maturity ratings of the breasts and pubic hair were Tanner stages 2 and 1, respectively. Bone age was 11 years, which was more than 2 years behind her chronological age. The skeletal survey was unremarkable except for a mild scoliosis. Biochemical tests revealed primary ovarian failure: follicle stimulating hormone (FSH) >190 mIU/mL (reference range (RR) 1.6–7); luteinizing hormone (LH) 50.3 mIU/mL (RR 1–7); estradiol <5 pg/mL (RR < 16). Other hormone levels were within normal range: insulin-like growth factor-1 (IGF-1) 325.66 ng/mL (RR 181–744); IGF-binding protein-3 (IGFBP-3) 2668.8 ng/mL (RR 1502–4427); prolactin 8.26 ng/mL (RR < 20); thyroid stimulating hormone (TSH) 7.7 μIU/mL (RR 0.5–4.5); free T4 1.65 ng/dL (RR 0.7–2.0) (Table S1). The results were normal for serum electrolyte, glucose, blood gases, hepatic and renal function, and routine urinalysis. Considering her severely short stature and growth deceleration, we performed a GH provocation test. The sampling for GH levels was carried out every 30 min for 120 min. The peak GH levels were 2.96 ng/mL, 3.63 ng/mL, and 3.06 ng/mL after the administration of arginine, L-dopa, and insulin, respectively. These results are indicative of complete GH deficiency.

**Figure 1:**
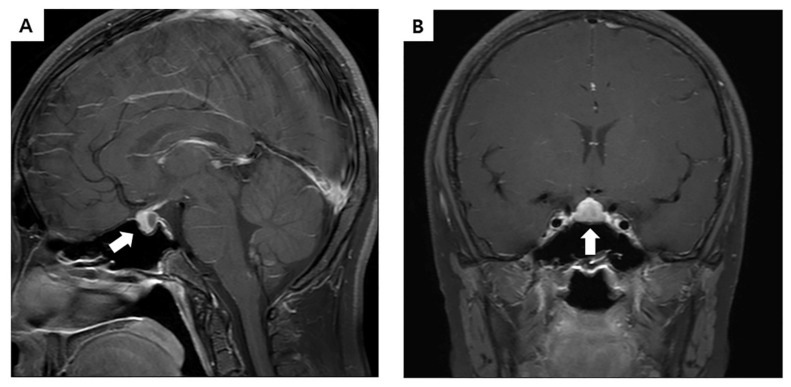
Sella magnetic resonance imaging (MRI) of the patient. T1 sagittal (**A**) and coronal (**B**) MRI showed a non-enhancing lesion in the posterior portion of pituitary gland measuring 8 mm in diameter with mild superior displacement of the optic chiasm (white arrow).

Sella magnetic resonance imaging (MRI) analysis revealed a non-enhancing pituitary adenoma measuring 8 mm in diameter with a mild superior displacement of the optic chiasm (Figure 1).

**Figure 2:**
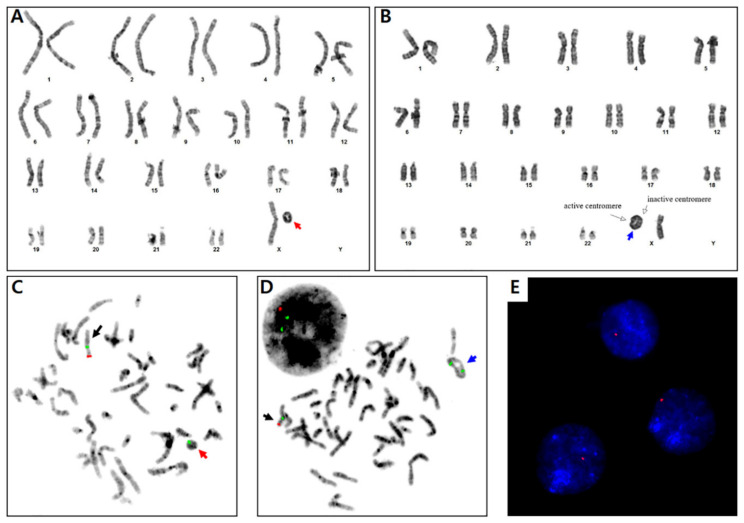
Chromosome study showed (**A**) a ring chromosome X (red arrow) in which breakage and reunion points are Xp22.2 and Xq23, and (**B**) a pseudodicentric ring chromosome X (blue arrow) with break and reunion at Xp22.2q27 and Xq25p11.2. Active centromere (Left white arrow) was on Xp22.2q27. (**C**,**D**) Metaphase fluorescent in situ hybridization (FISH) using an LSI KAL (on Xp22.3)/CEP X (on Xp11.1-q11.1) probe (Vysis, Dual Color Probe) showed a normal X chromosome (black arrow; one green and one red signal), a ring chromosome X with loss of the *KAL* gene (one green signal), and a pseudodicentric ring chromosome X with loss of *KAL* (two green signals). (**E**) Interphase FISH study using a TelVysion Xq/Yq probe (Vysis, Single Color probe) showed a single Xq telomere signal (red), indicating Xq telomere loss in each cell. Magnification, ×400.

A conventional chromosome study using peripheral blood showed the 98/177 (55.4%) cells with ring chromosome X, 75 (42.4%) cells with monosomy X, and 4 (2.2%) cells with pseudodicentric ring chromosome X: mos 46,X,r(X)(p22.2q23)(98)/45,X(75)/46,X,psu dic r(X;X)(p22.2q27;q25p11.2) [4] (Figure 2A,B), which indicated a mosaic TS. The subsequent fluorescence in situ hybridization (FISH) using an LSI KAL/CEP X probe (Vysis, Abbott Molecular Inc.) and a TelVysion Xq/Yq probe (Vysis) showed that r(X) lacked the *KAL* (*ANOS1*) gene on Xp22.3 and the Xq telomere (Figure 2C–E).

The results of the renal ultrasonography and echocardiography were normal. To evaluate the possibility of other pituitary hormone deficiencies, a combined pituitary stimulation test (i.e., the cocktail test) was performed; decreased cortisol (peak cortisol 12.2 μg/dL; RR > 22 μg/dL) secretion was observed following insulin-induced hypoglycemia, which indicates adrenocorticotropic hormone (ACTH) deficiency (secondary adrenal insufficiency). The patient was administered maintenance physiologic doses of hydrocortisone, and recombinant human GH therapy was also initiated. The initiation of estrogen replacement therapy will be determined by the patient’s growth velocity and emergence of secondary sexual characteristics.

## 3. Discussion

TS is associated with a constellation of potential abnormalities involving numerous organ systems, making it a challenging disorder for health care providers and families. Short stature, one of the common presentations that pediatricians encounter in clinical practice, is a clinical hallmark of TS. Nearly 5% of children referred for an evaluation of short stature have an identifiable pathologic cause, such as GH deficiency, chronic disease, or a genetic condition (e.g., TS) [9,10].

TS and GH deficiency are important differential diagnoses in females with short stature and are the two most frequently approved conditions for GH treatment [11]. TS can be differentiated from GH deficiency by delayed bone age, hypogonadism, characteristic phenotypic features, and peak GH levels after GH provocation tests [12]. Approximately 60% of TS may not have marked stigmata of the syndrome, such as webbed neck, wide-based nipples, and wide carrying angle to the arms, especially in girls with Turner mosaicism [13]. Short stature and delayed puberty may be the only symptoms of TS. However, other physical abnormalities may also be variably expressed. Our case showed delayed bone age and breast development, which are not common symptoms of TS. This emphasizes the importance of chromosomal analysis to rule out TS in girls with short stature [9]. Furthermore, it is important to check for GH deficiency by provocation tests in TS patients with retarded growth rates by a height of less than the 3rd percentile for their age and sex.

The coexistence of GH deficiency and TS is a very rare condition. To our knowledge, there are only a few reported cases of TS associated with GH deficiency (Table 1) [12,14,15,16,17]. Pituitary adenomas have also rarely been identified in TS patients. Review of the literature demonstrated nine case reports of women with TS who presented with pituitary adenomas during late adolescence or adulthood; six were diagnosed with functioning pituitary adenoma and three with non-functioning pituitary adenoma as in our case [8,18,19,20,21,22,23,24,25] (Table 2). Non-functioning pituitary adenomas in children and adolescents are rare; they comprise only 4 to 6% of pediatric patients, while they account for approximately 33 to 50% of adult patients with pituitary lesions [26,27,28]. This case presented with non-functioning pituitary adenoma associated with GH and ACTH deficiency, which is in accordance with a previous study demonstrating that non-functioning pituitary adenomas may present with GH deficiency (up to 75%), LH/FSH deficiency (~40%), or ACTH and TSH deficiency (~25%) [29].

The incidental pituitary adenoma with the co-occurrence of GH deficiency and TS is very rare, and there have been no other reported cases of the co-occurrence of pituitary microadenoma, GH deficiency, and TS; the causal relationship is difficult to explain. This case demonstrates that investigating the underlying causes of short stature should be primarily based on clinical presentations and physical examination, while an accurate diagnosis is made through a combination of clinical, biochemical, and radiological evaluations.

This case was cytogenetically characterized with a unique mosaicism for three types of cells with r(X), monosomy X, and psu dic r(X), which may have occurred as a process of dynamic mosaicism. The amount of Xq deletion and r(X) has been known to associated with phenotypic severity [30]. In particular, the presence of the *XIST* gene on Xq13.2 is important. *XIST* located in the X-inactivation center is essential for the initiation and spread of X chromosome inactivation. As a general rule, when one X chromosome is structurally abnormal without involving an autosome, it is typically inactivated in a majority of cells [31,32]. However, an abnormal X chromosome that lack *XIST* fails to become inactivated, which may be associated with a more severe phenotype, including mental retardation. Fortunately, r(X) observed in our patient had intact Xq13 including *XIST*, therefore, it was expected to be inactivated. This case had a mild Turner variant phenotype, without any cardiac defect, renal malformation, and low intelligence. However, the biochemical findings revealed elevated LH and FSH levels, suggesting primary ovarian failure [33]. The patient also revealed multiple pituitary hormone deficiencies with pituitary adenoma. Given such complex phenotypes of this patient and that mosaic levels decrease with age due to the vulnerable character of r(X) [34], it is challenging to identify the effect of mosaicism on clinical phenotypes.

Recombinant human GH is a standard treatment for TS patients, although physiologically significant alterations in GH secretion have not been identified [1]. Short stature in TS is not due to hormonal deficiencies but is a consequence of haploinsufficiency of the short stature homeobox gene located on the short arm of the X chromosome (*SHOX*), a transcriptional activator in the osteogenic cell line [35]. The *SHOX* gene is located in the critical region on the X chromosome that escapes X-inactivation, and mutations or deletions are likely to exert a dosage effect [36]. When *SHOX* haploinsufficiency occurs, there is decreased chondrocyte proliferation and differentiation at the growth plate, leading not only to short stature, but also skeletal abnormalities [37]. GH stimulates linear bone growth and acts at the epiphysis to promote prechondrocyte differentiation and osteoblast expansion [1]. Promptly initiating treatment would enable the affected patients to reach an adult height within the normal population range [38,39].

In conclusion, this paper reports the first case of a unique mosaic TS patient with GH deficiency and pituitary adenoma. This case broadens and further delineates the complex genotype–phenotype of TS, and highlights the importance of performing a thorough, multidisciplinary assessment that considers numerous potential diseases and concomitant conditions when evaluating patients with short stature.

## Figures and Tables

**Table 1:** Comparison of clinical and laboratory features between this case and previously reported cases of TS associated with GH deficiency.

	Case in This Study	Yu et al. [12]	Yu et al. [12]	Brook et al. [13]	Efstathiadou et al. [14]	Gallicchino et al. [15]	Jin et al. [16]
Age at diagnosis (y)	12.3	8.9	12.3	9.1	30	11	11
Turner syndrome	12.3	7.5	12.3	9.6	17	12	11
GH deficiency							
Height (SDS) at diagnosis	−3.4	−1.89	−1.72 ^†^	−3.6	−2.35	−4.2	−3.69
Turner syndrome	−3.4	−2.30	−1.72 ^†^	NA	−6.0	−4.9	−3.69
GH deficiency							
Karyotype	46,X,r(X)/45,X/46,X,psu dic r(X;X)	45,X/45,X+mar	45,X/46,XX	45,X	45,X	45,X/46,XX	45,X
Peak GH on GH provocation test (ng/mL)	3.63	6.17	7.38	6.1	4.65	0.14	<5
Other pituitary hormone deficiencies	ACTH	None	None	None	TSH, gonadotropin	TSH, gonadotropin	None
Associated conditions	Subclinical hypothyroidism, pituitary microadenoma	Partial empty sella, horseshoe kidney	None	None	None	Empty sella	Chronic lymphocytic thyroiditis

GH: growth hormone, SDS: standard deviation scores, TSH: thyroid stimulating hormone, NA: not available. ^†^ Height after 2 years of growth hormone therapy.

**Table 2:** Comparison of clinical and laboratory features between this case and previously reported cases of TS associated with pituitary adenomas.

	Case in This Study	Yeh et al. [8]	Bolanowski et al. [18]	Gaspar et al. [19]	Mermilliod et al. [20]	Weibel et al. [21]	Dotsch et al. [22]	Willemse et al. [23]	Yamazaki et al. [24]	Gelfand et al. [25]
Age at TS diagnosis (yr)	13	16	10	16	16	43	12	19	33	26
Age at pituitary disease diagnosis (yr)	13	16	33	25	18	43	19	26	33	29
Karyotype	46,X,r(X)/45,X/46,X,psu dic r(X;X)	45,X	45,X/46,X,i(X) (q10)	45,X/46,XX	45,X	45,X/46,XX/47,XXX	45,X/46,XX	45,X	47,XXX/45,X/46,XX	45,X/47,XXX
Symptoms or labs related to pituitary disease	Short stature	Headache, vomiting, cranial nerve IV palsy	Facial changes, increased hand/foot size	Secondary amenorrhea, galactorrhea	Hypogonado-tropic hypogonadism	Unexpected normalization of FSH level	Secondary amenorrhea, hyperprolactinemia	Change in appearance, enlarged feet	Dysphagia due to soft palate edema, enlarged hands/feet	Weight gain, ankle edema, acne, hirsutism
Pituitary hormone abnormalities	Deficiency in GH, ACTH	Deficiency in GnRH	GH excess	Prolactin excess	Deficiency in GnRH	Deficiency in GnRH	Prolactin excess	GH excess	GH excess	Cortisol excess

## Supplementary Materials

Supplementary materials can be found at https://www.mdpi.com/2075-4418/10/10/783/s1.
