# Supplementary material for: Coexistence of Growth Hormone Deficiency and Pituitary Microadenoma in a Child with Unique Mosaic Turner Syndrome: A Case Report and Literature Review
Source: Diagnostics (Basel). 2020 Oct 4;10(10):783. doi: 10.3390/diagnostics10100783 (PMC7600578; doi:10.3390/diagnostics10100783)
Supplement: Supplementary file 1 [file diagnostics-10-00783-s001.pdf]

Table S1.Results of biochemical tests.

	Results (reference)
FSH (mIU/mL)	>190 (1.6 – 7)
LH (mIU/mL)	50.3 (1 – 7)
Estradiol (pg/mL)	<5 (<16)
IGF-1 (ng/mL)	325.66 (181 – 744)
IGFBP-3 (ng/mL)	2668.8 (1502 – 4427)
Prolactin (ng/mL)	8.26 (<20)
TSH (μIU/mL)	7.7 (0.5 – 4.5)
Free T4 (ng/dL)	1.65 (0.7 – 2.0)

FSH: follicle stimulating hormone, LH: luteinizing hormone, IGF-1: insulin-like growth factor-1, IGFBP-3: insulin-like growth factor-binding protein-3, TSH: thyroid stimulating hormone.

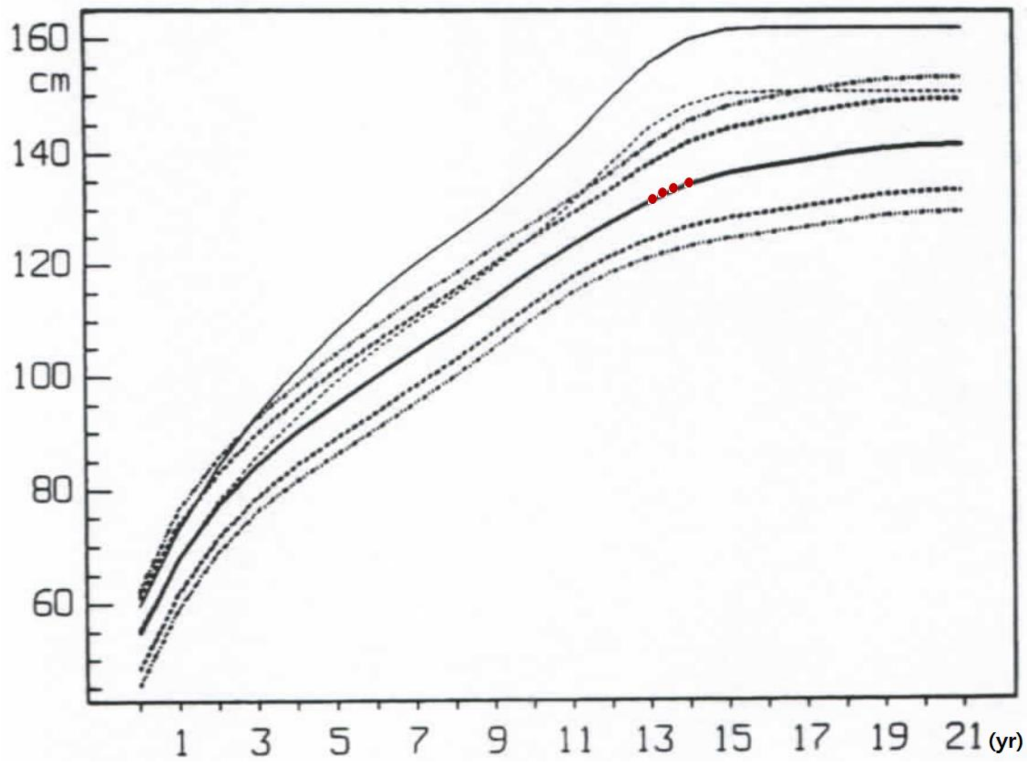

Figure S1. The height of our patient (red dots, 50th percentile) in growth curves for girls with Turner syndrome. Data derived from Bernasconi et al [1].

Reference

Bernasconi, S.; Larizza, D.; Benso, L.; Volta, C.; Vannelli, S.; Milani, S.; Aicardi, G.; Berardi, R.; Borrelli, P.; Boscherini, B.; et al. Turner's syndrome in Italy: familial characteristics, neonatal data, standards for birth weight and for height and weight from infancy to adulthood. *Acta Paediatr*. **1994**, *83*:292-298.
